# Image-Based Numerical Analysis for Isolated Type II SLAP Lesions in Shoulder Abduction and External Rotation

**DOI:** 10.3390/diagnostics13101819

**Published:** 2023-05-22

**Authors:** Javier A. Maldonado, Duvert A. Puentes, Ivan D. Quintero, Octavio A. González-Estrada, Diego F. Villegas

**Affiliations:** 1 Department of Mechanical and Mechatronics Engineering, University of Waterloo, 200 University Avenue West, Waterloo, ON N2L 3G1, Canada; jamaldon@uwaterloo.ca; 2 School of Mechanical Engineering, Universidad Industrial de Santander, Carrera 27 Calle 9, Bucaramanga 680002, Colombia; duvert_098@hotmail.com (D.A.P.); agonzale@uis.edu.co (O.A.G.-E.); 3 School of Medicine, Universidad Industrial de Santander, Carrera 27 Calle 9, Bucaramanga 680002, Colombia; ivanquinterop@gmail.com

**Keywords:** isolated type II SLAP, biomechanics, glenohumeral joint, nonsurgical treatment, computational biomechanics

## Abstract

The glenohumeral joint (GHJ) is one of the most critical structures in the shoulder complex. Lesions of the superior labral anterior to posterior (SLAP) cause instability at the joint. Isolated Type II of this lesion is the most common, and its treatment is still under debate. Therefore, this study aimed to determine the biomechanical behavior of soft tissues on the anterior bands of the glenohumeral joint with an Isolated Type II SLAP lesion. Segmentation tools were used to build a 3D model of the shoulder joint from CT-scan and MRI images. The healthy model was studied using finite element analysis. Validation was conducted with a numerical model using ANOVA, and no significant differences were shown (*p* = 0.47). Then, an Isolated Type II SLAP lesion was produced in the model, and the joint was subjected to 30 degrees of external rotation. A comparison was made for maximum principal strains in the healthy and the injured models. Results revealed that the strain distribution of the anterior bands of the synovial capsule is similar between a healthy and an injured shoulder (*p* = 0.17). These results demonstrated that GHJ does not significantly deform for an Isolated Type II SLAP lesion subjected to 30-degree external rotation in abduction.

## 1. Introduction

The shoulder complex is the major joint in the upper body. It comprises five joints that act together to allow upper limb mobility [1,2]. One of these is the glenohumeral joint (GHJ), which is the most prone to suffer injuries and cause instability pathologies [3]. Superior labral anterior to posterior (SLAP) lesions involve the soft tissues of the GHJ, specifically, the upper edge of the glenoid components. Moreover, they can induce chronic pain and decreased stability of the GHJ in active persons [4]. Possible causes of these lesions are hyperextension falling on the outstretched extremity, heavy lifting, and direct trauma [5]. Overhead athletes are the most prone to suffer SLAP tears [6]. The SLAP tear is classified into four subtypes depending on the extent of the labral tear and biceps anchor damage [7]. However, it has been further subdivided to delineate ten different types of SLAP tears [8,9]. Type I is defined as the degeneration of the tissue, which is generally asymptomatic. Type II is a complete detachment between the labrum and humeral glenoid, and it is generally diagnostic by both pain and instability. Type III and Type IV are defined as bucket handle tears in the central portion of the structure. The difference between Type III and Type IV is that the long tendon of the bicep does not have any lesion in Type III, whereas the bicep tendon is displaced in Type IV. Type V is a mix between SLAP and Bankart lesion, and Type VI is based on an unstable labrum. Type VII is a SLAP lesion that involves the Medium Glenohumeral Ligament (MGHL), and Type VIII is a Type II SLAP that extends until the inferior posterior region. Type IX SLAP lesion is characterized as a complete labral injury that extends throughout the entire circumference of the glenoid. A Type X SLAP lesion refers to a tear of the superior labrum, which is also accompanied by a posterior-inferior labral tear, commonly known as a reverse Bankart lesion.

On the other hand, Snyder et al. [10] indicated that Isolated Type II SLAP tear is the most common type of SLAP lesion, involving about 55% of the total diagnosed lesions. It is well-known that non-operative management is the first option for patients with a SLAP lesion [11]. However, surgical treatment is needed when nonsurgical intervention fails. The three most common options for surgical treatment in an Isolated Type II SLAP are arthroscopic SLAP repair, biceps tenodesis alone [12], or biceps tenotomy [13]. There is still a controversy about which surgical technique is preferred and if surgical treatment is the best option [14,15]. Although some authors support the fact that nonsurgical treatment is unsuccessful [7], others promote nonsurgical treatment [16,17]. Return-to-play for overhead athletes ranged from 22% to 94%, and recent research shows around 64% success in nonsurgical treatment [18]. SLAP Type II lesions are typically classified into three subtypes: (i) an anterosuperior type II SLAP lesion, (ii) a posterosuperior type II SLAP lesion, and (iii) a combined anterior and posterior type II SLAP lesion [19].

In this sense, a computational approach may support treatment for Isolated Type II lesions based on the results of the biomechanical models. For example, several studies have used finite element analysis (FEA) to evaluate the labrum effect over capsular strain distribution [20,21,22]. Moreover, some authors have developed kinetic models of the shoulder to evaluate the instability lesions from the labrum without a capsule, studying the influence of geometrical parameters of the humeral head on the mechanics of cuff tear arthropathy [23,24]. However, no studies have assessed the effect of an Isolated Type II SLAP lesion. Furthermore, the effect of SLAP lesions in isolated regions can be investigated by determining the principal maximum strain in each region.

The present study aims to evaluate the mechanical response of the soft tissues on the glenohumeral joint with an Isolated Type II SLAP lesion of Snyder classification, defined as a slight tear between the upper part of the labrum and the glenoid components. The objective is to clarify the biomechanics of the Isolated Type II SLAP lesion subjected to 30-degree external rotation in abduction by comparing the biomechanical performance of a healthy shoulder against a shoulder with SLAP Isolated Type II, considering the three subtypes.

## 2. Methods

### 2.1. 3D Model

A 3D model of the GHJ was conducted using computational segmentation from CT scans and MRI images. The patient was a 23-year-old Hispanic male, with a height of 174 cm, without previous shoulder pathologies. We assume symmetry of the biomechanical conditions; thus, only one shoulder is studied [25]. DICOM files were used for obtaining the 3D model of bones using the imaging segmentation software 3D Slicer v4.11 [26], applying semi-automatic segmentation. The slide thickness for the image was 0.7 mm, 0.5 mm, and 0.4 mm in sagittal, transverse, and coronal, respectively. Then, the model was processed with the CAD software SolidWorks v29 to prepare the geometry. The capsular joint was manually reconstructed using borders from the MRI, insertion lines from CAD, and theoretical data, including synovial cavity thickness, tendon thickness, and insertion zones [1]. The GHJ acts as having rotation without translation on some axes of the glenohumeral head [1]. In addition, the capsule was built to preserve the insertion area of the soft tissues defined in the CAD model.

### 2.2. Surface Reconstruction

The natural axes of rotation of the humerus were generated to represent movements with greater anatomic precision. The metaphyseal cylinder was connected to the epiphyseal, which was generated as a sphere. The center of this sphere was set as the center of rotation of the humerus, and the longitudinal axis of the cylinder was set as the humerus axis. In addition, an offset ratio table with an epiphyseal sphere radius was interpolated to obtain the actual offset of this model [27].

The retroversion axis was found perpendicular to the center of the junction line of the ends of the cartilage, in a cutting plane that coincides with half of the sphere from the transversal view. Then, the bicipital distance (*BD*) was evaluated as the perpendicular distance between the retroversion axis and the end of the tuberosity of the humeral head. Equation (1) shows the retroversion angle [28].

(1) Retroversion=−2.33BD[mm]−0.1

**Figure 1:**
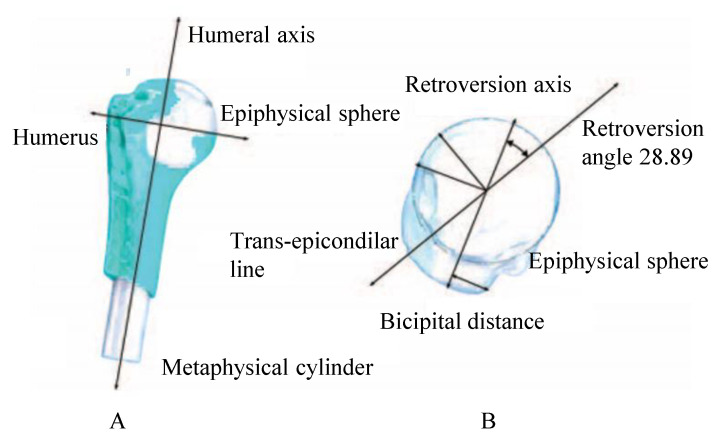
(**A**) Humerus presenting the epiphyseal sphere insertion and the metaphyseal cylinder front view, (**B**) model top view.

Then, the trans-epicondylar axis was drawn from one end of the humeral head to the other. This axis must have an angle concerning the retroversion axis equal to the retroversion angle. A bicipital distance of 12.1 mm and retroversion of −28.89° were calculated, the negative sign means an angle in the posterior direction. All anatomical axes are shown in Figure 1. With these data, the axes were drawn in the CAD model, having the axis of retroversion parallel to the axis perpendicular to the face of the glenoid component [29]. Finally, another line was drawn on the face of the glenoid component that crosses the center point of the same [30].

**Figure 2:**
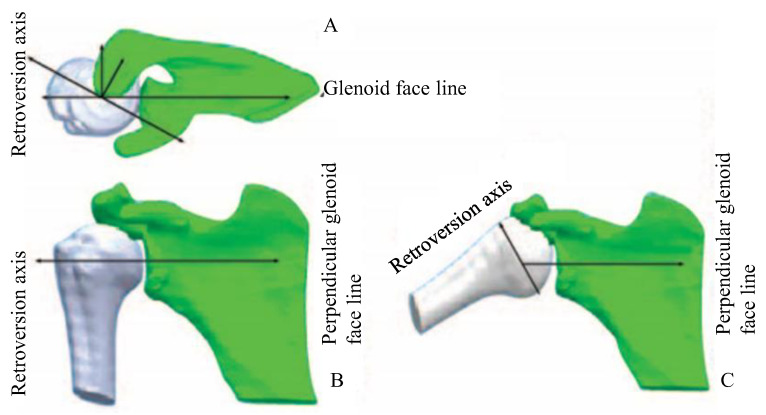
(**A**) Shoulder in the neutral position on the top view, (**B**) shoulder in the neutral position on the front view, (**C**) shoulder bones in the initial position on the front view.

**Figure 3:**
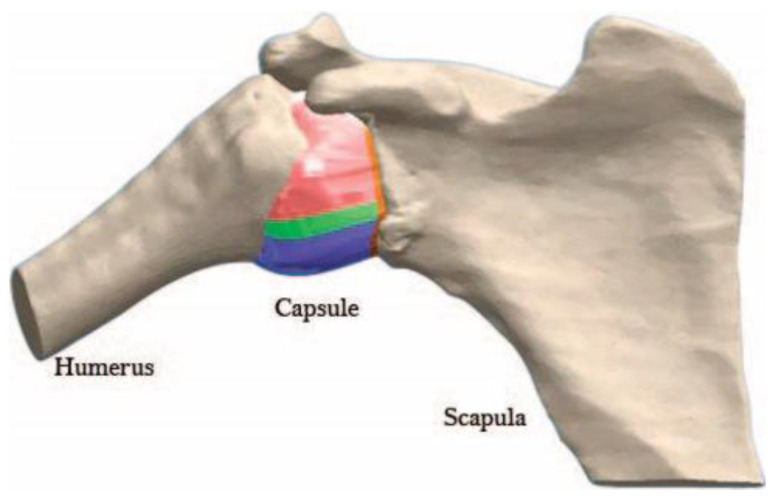
Shoulder with the capsule divided into five sections according to their material.

The structure was taken to the initial position, defined as a humerus at 90° of abduction, 0° elevation, and 0° of external rotation. Notice that due to the relative movement of the scapula, the 90° of the abduction of the humerus is 60° of inclination against the glenoid component [29], as shown in Figure 2. The capsule was reconstructed according to the measures of the regions in the patient’s data, as shown in Figure 3. A surface of 4 mm of thickness is included at the edge of the capsule, between the capsule and bones, to represent the labrum [21].

**Figure 4:**
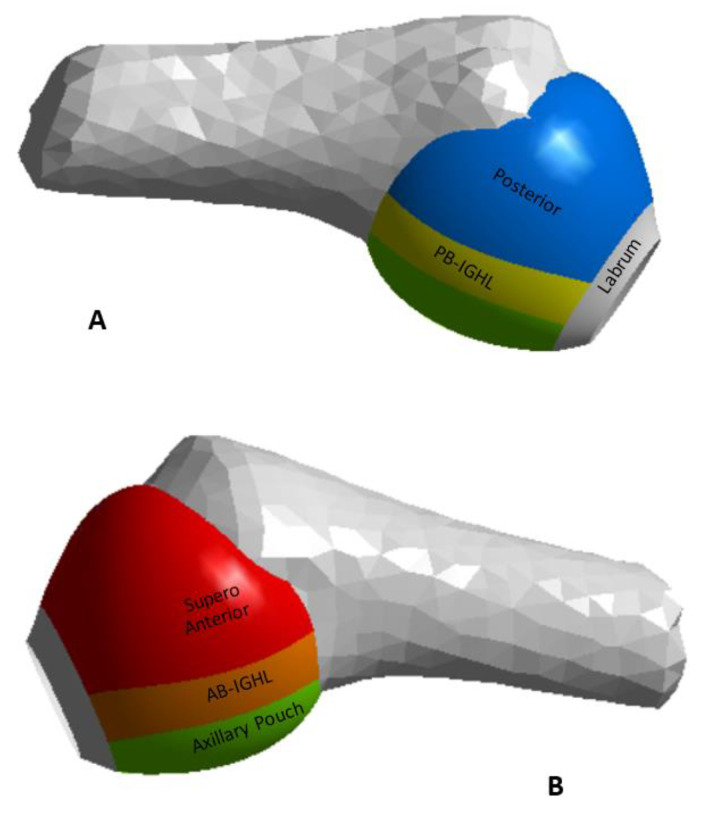
(**A**) Glenohumeral capsule posterior view, (**B**) glenohumeral capsule anterior view.

The capsule was divided into five regions, and their limits were determined based on the surface grooves of the GH ligaments, where both structures work like one flexible body. Differences in biomechanical properties and thickness characterize these regions: (i) the anterior band of the inferior glenohumeral ligament (AB-IGHL), (ii) the axillary pouch (AP), (iii) the posterior band of the inferior glenohumeral ligament (PB-IGHL), (iv) the super anterior capsule, and (v) the posterior capsule [31]. The CAD model with the sections is shown in Figure 4. The thickness reported in previous studies was used for each section, including the labrum [20].

## 3. Materials

Several models of glenohumeral articulation have been presented in the literature, considering elastic [21] and hyperelastic [30] isotropic materials. In general, hyperelastic isotropic materials adjust better to the mechanical behavior of soft tissues of the glenohumeral joint [20,21]. Therefore, the Veronda–Westmann and the Yeoh models for hyperelastic isotropic material were considered based on this.

Hyperelastic materials are defined by their strain energy density function *W*, which can be expressed in terms of the invariants of the deformation. For an incompressible isotropic material, three invariants are defined by the principal stretches λ according to Equation (2). Additionally, the principal Cauchy stress $\sigma_{1}$ under uniaxial tension, with $\sigma_{2}=\sigma_{3}=0$, is obtained from the derivative of the strain energy density concerning the invariants, as shown in Equation (3).

(2) $$I_{1}=\lambda^{2}+\frac{2}{\lambda };\;I_{2}=2\lambda +\frac{1}{\lambda^{2}};\;I_{3}=1$$

(3) $$\sigma_{1}=\frac{\partial W}{\partial \lambda }=2\left(\lambda^{2}+\frac{2}{\lambda }\right)\left(\frac{\partial W}{\partial I_{1}}+\frac{1}{\lambda }\frac{\partial W}{\partial I_{2}}\right)$$

### 3.1. Yeoh Model

The Yeoh model, which is often expressed in third order, is a phenomenological model characterized by using only a polynomial base and the first invariant diverter of stress [32]. Equation (4) shows the strain energy density function, where $C_{10}$, $C_{20}$, and $C_{30}$ are material constants for a third-order polynomial base [33].

(4) $$W=C_{10}\left(I_{1}-3\right)+C_{20}{\left(I_{1}-3\right)}^{2}+C_{30}{\left(I_{1}-3\right)}^{3}$$

Table 1 shows the values for the coefficients used in the Yeoh model for the different regions of the joint capsule. These values were evaluated using least squares fitting from the Veronda–Westmann constants provided in [30] for the glenohumeral ligaments with capsule, with the coefficient of determination $r^{2}>0.97$. The properties for the labrum are used 45.6 MPa as tensile modulus [21], and 0.449 of Poisson’s ratio [20].

### 3.2. Finite Element Model and Boundary Conditions

**Figure 5:**
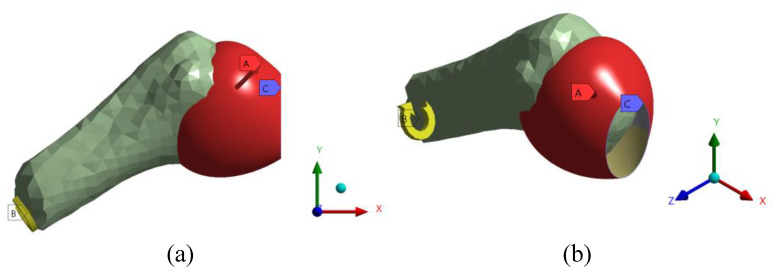
(**a**) Front view, and (**b**) isometric view for the boundary conditions applied to the 3D model: uniformly distributed pressure caused by the synovial capsule (A), external rotation applied on the humerus in the axial direction (B), and labrum fixed boundary (C).

Modeling was conducted using the general-purpose finite element software ANSYS v2020R1 [34]. The FE model analyzed the capsule as a structure composed of five regions according to ligaments, as proposed by [21]. Thus, the mechanical behavior of each ligament can be calculated independently. The capsule was considered a surface and meshed with SHELL181 elements, using triangular elements for irregular geometries and quadrilateral elements for regular geometries where structured meshes can be produced. The humerus was considered a rigid body meshed with 7836 elements. The scapula was considered a fixed structure to study the relative displacement of the humerus [21,35,36]. A rotation in the humerus was applied in an axial direction from 0 degrees to 30 degrees to simulate an external rotation test [21], as shown in Figure 5. In addition, an internal pressure of 0.7 KPa was applied to the capsule to represent the internal pressure produced by the synovial capsule [30,37,38].

The finite element model was based on a simple stability test which consists of compression and external rotation of the humerus [39]. Tendons were not included, and the study focused on the biomechanical behavior of the joint capsule and their ligaments. The compression test was selected with simple rotation because of its low false positive rate and its high rate of true positives [40]. A mesh independence test was performed to guarantee asymptotic convergence of the equivalent elastic strain, for a final element size of 2 mm. Then, the values of maximum principal strain in the main regions were determined to perform the validation of the nonpathological model.

Bonded contact conditions were defined between the insertion line of the capsule and the humeral head, the glenoid components bonded along the edges, and a frictionless contact between the humeral head and the inner surface of the synovial capsule. Once the biomechanical calculations of the glenohumeral articulation without pathologies were conducted, validation was made. One-way ANOVA was performed in each tendon to compare the maximum principal strains for the 3D model used in the present study and the 3D model used in a previous study [21]. We used an odd number of samples to ensure no data repetition, and the statistical power was defined as 80%. In addition, the significance level was 5%. The two models had no significant differences (*p* = 0.47) for the AB-IGHL section. Then, the model is modified to represent the Isolated Type II SLAP lesion. Finally, maximum principal strains were obtained for an injured and healthy shoulder.

### 3.3. A Joint Model with an Isolated Type II SLAP

**Figure 6:**
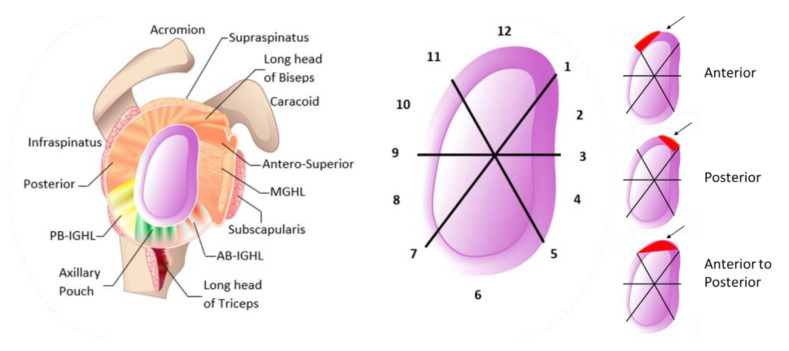
The glenoid labral division in time zones used to define subtypes of SLAP lesions. From 12 to 1: anterior SLAP, from 11 to 12: posterior SLAP, and from 11 to 1: anterior to posterior SLAP.

SLAP lesions are prevalent, accounting for as much as 26% of injuries detected during shoulder arthroscopy. Among them, Isolated Type II lesions are the most widespread [5,41,42]. Based on this pathological model, the numerical model was defined using the same contact, displacements, and boundary conditions. The Isolated Type II lesion of the model was built using data from medical images and CAD software. Labrum and glenoid component tears were simulated by changing the fixed support ratio of the insertion zone. First, the entire insertion area was fixed, and then, the contact was eliminated in the superior-anterior zone. Consequently, there were simulated examples of the three subtypes of Isolated Type II SLAP lesions [19], this kind of lesion can be determined with the glenoid labral division [43]. Figure 6 shows how the glenoid can be divided into clock zones, and the labrum is compared to a clock face where its superior section is situated at 12 o’clock and its inferior section at 6 o’clock [44], where anterior SLAP is a lesion from 12 to 1, posterior SLAP is a lesion from 11 to 12, and anterior to posterior SLAP is a lesion from 11 to 1.

## 4. Results

The process of obtaining and defining an aligned 3D model of the GJ was based on previous studies [45,46,47]. It allowed the calculation of the retroversion without the epicondyle, the anatomical structure used to measure the retroversion angle from the humeral head [48]. Maximum principal strains of the healthy model show differences of 19%, 3%, and 16% in AB-IGHL, axillary pouch, and PB-IGHL, respectively. These results agree with the work by Drury [21]. The highest strain was shown in the lower regions of the capsule AB-IGHL (28.6%) at 30-degree external rotation movement in abduction. It was followed by the axillary pouch (25.2%), which helps withstand rotation and anterior displacement. In addition, the posterior band of the glenohumeral (17.8%) ligament exhibited less strain due to its function to limit the translation when this movement is performed.

**Figure 7:**
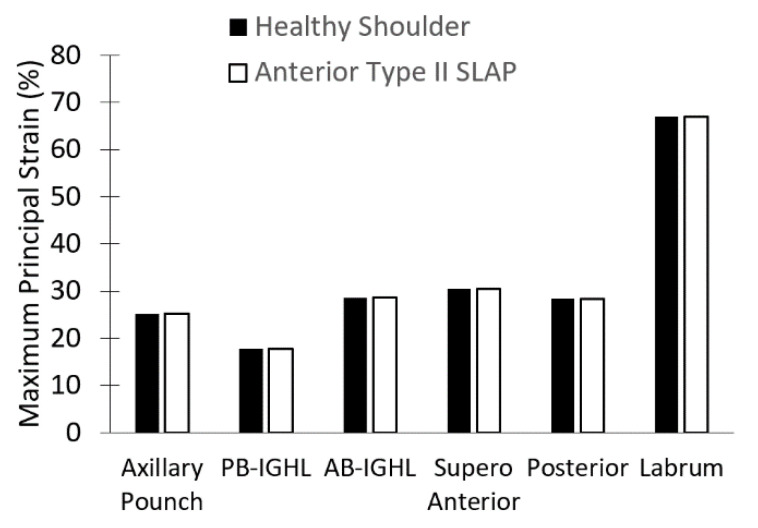
Maximum principal strain for the healthy glenohumeral joint and an injured joint (Isolated Type II SLAP lesion) at 30° with an anterior Isolated Type II SLAP.

**Figure 8:**
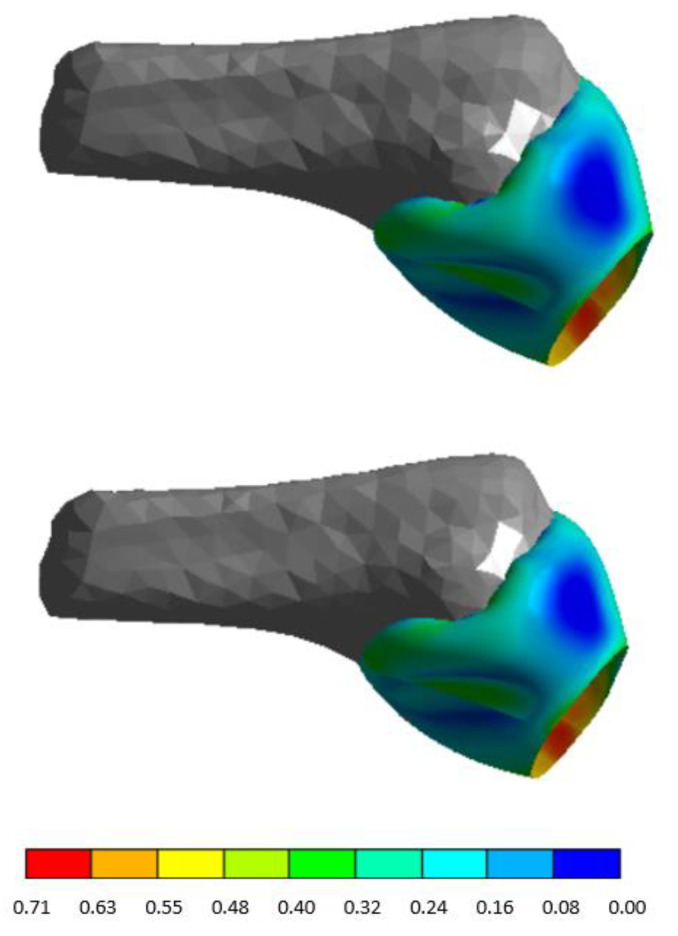
Maximum principal strain distribution for the healthy glenohumeral joint and injured joint (Isolated Type II SLAP lesion) at 30° with an anterior Isolated Type II SLAP. The other three lesion cases have the same performance.

The four models, the healthy glenohumeral joint and the three Isolated Type II SLAP lesions, allowed a comparison of the strain distribution in the anterior bands. Three pathological cases obtained the same results. Consequently, the anterior Type II SLAP was used as a reference. Notice the strain in both cases is slightly similar (*p* = 0.17), as seen in Figure 7 and Figure 8. The detachments and their surrounding area are the only exceptions, but they do not affect the rest of the structure.

## 5. Discussion

No previous studies address the three subtypes of Isolated Type II lesions from the computational point of view. Therefore, this work aimed to determine how the soft tissue of the GHL responds to a rotation of 30°. Previous computational models have assessed the response of the bone structure to SLAP lesions but not the response of the soft tissue [23].

Maximum principal strain results of the healthy shoulder model suggested that the anterior band of the inferior glenohumeral ligament is a region of high strain (28.6%) for small rotations (up to 30°). Considering its small size with slightly same properties compared to the axillary pouch or the anterosuperior region, it is the most prone region to injury [49]. The lowest maximum principal strains were shown in the PB-IGHL (17.8%). Furthermore, the translation prefers the anterior direction due to the external rotation movement, and the axillary pouch also has an important role in the shoulder movement holding the humeral head. On the contrary, the PB-IGHL band showed a strain to hold it in an elongated position during rotation. Therefore, this computational model supports previous results showing that 95% of dislocations occur in the anteroinferior direction [2].

Moreover, the results confirm the role of the labrum in the movement of the glenohumeral joint due to maximum principal strains increased by up to 70% for only 30 degrees of external rotation in abduction. In addition, the capsule can maintain the stability of the entire joint despite the anterior SLAP. The model evaluated the strains for 30 degrees of external rotation in abduction where the biceps have maximum participation [50], and the authors expect a similar behavior for higher rotations considering the relation between the biceps tendon and SLAP [51].

The results of this study are significant because the similarity in strains between the healthy and lesion models suggests a minimum involvement of the upper section of the capsule for small external rotations in abduction. The capsular structure can carry out an external rotation without adverse effects on the shoulder up to 30 degrees from the biomechanics point of view. This result can be associated with using external rotation movement at 90 degrees in abduction as a recommended exercise for the nonsurgical treatment of SLAP lesions, allowing the strengthening of muscles without hurting the capsule [42,52]. Further studies could assess higher rotations to determine the response of the capsule. Usually, baseball players lead the shoulder in high external rotations; thus, computational models can be used to validate nonsurgical treatments [53]. Moreover, a relevant percentage of baseball players health the injure with nonsurgical treatments (32 of 44) [53]. Despite this, it is notorious how the nonsurgical treatment for SLAP is a tentative alternative, with some aspects to improve. Variations in both the therapy movements and the safe angles for these movements can expand options for health professionals [42,50,52].

The similarity of the results between the healthy and the pathology model suggests that the role of the upper section of the glenoid is minimum for small external rotations. Therefore, other movements should be studied to clarify whether the SLAP can be treated only with nonsurgical therapy. For that reason, it is suggested to model numerically and experimentally other stability tests, such as the Hawkins–Kennedy test, which will help clarify the involvement of the posterior band during a SLAP. Surgeons could use the results obtained in this study to help decide on the most appropriate treatment for an Isolated Type II SLAP lesion.

This study has some limitations. The bicipital tendon was not included in the computational model for the sake of simplicity. However, the detachment of the labrum is applied in the insertion area of the long head of the bicipital tendon. From this point of view, adding a body to the model to simulate the tendon behavior in the test is recommended. The tendon could be represented as a body with a load collected from experimental data [36]. In addition, some values varied between this study and the studies in [21,22], probably due to geometrical differences between models. These differences are the humeral retroversion and the capsule size of the patient. However, how each band operates concerning the others is preserved. In other words, the axillary pouch is the region that showed more strain compared to the others, and the posterior region has the lowest maximum principal strain. Moreover, results could vary with the use of different models, as only the shoulder segmentation of an average Hispanic male was utilized. Considering the assumptions and limitations of the model, there is a need for subject-specific studies and models with higher degrees of rotation for future work.

## 6. Conclusions

In summary, an isolated SLAP does not generate a significant change in the biomechanical performance of the shoulder, and no negative effects are expected at 30 degrees of external rotation in abduction for anterior Isolated Type II SLAP lesions. Therefore, external rotation movement can be considered an appropriate exercise for the nonsurgical treatment of athletes who carry out movements with small external rotations and workers who do not carry weights above their height. Results do not differ considerably from the non-operative treatment [54], and nonsurgical measures or surgery are recommended even for failed SLAP repair [42]. The results obtained in this study will also help with upgrading surgical algorithms for SLAP lesions proposed by some authors [11].

## Figures and Tables

**Table 1:** Material constants in the Yeoh model for the different regions of the joint capsule.

Region	C1 [MPa]	C2 [MPa]	C3 [MPa]	R^2^	Thickness [mm]
Antero Superior	20.5	−26.5	185.3	0.99	2.8
Posterior	13.2	−19.6	125.9	0.99	1.5
AB-IGHL	11.9	−11.1	95.8	0.99	2.8
PB-IGHL	29.1	−18.4	206.5	0.99	1.3
Axillary pouch	0.22	−17.4	111.9	0.99	4.3

## Data Availability

The data presented in this study are available on request from the corresponding author.
